# Unipolar (Dendritic) Brush Cells Are Morphologically Complex and Require Tbr2 for Differentiation and Migration

**DOI:** 10.3389/fnins.2020.598548

**Published:** 2021-01-08

**Authors:** Ashley McDonough, Gina E. Elsen, Ray M. Daza, Amelia R. Bachleda, Donald Pizzo, Olivia M. DelleTorri, Robert F. Hevner

**Affiliations:** ^1^Center for Integrative Brain Research, Seattle Children’s Research Institute, Seattle, WA, United States; ^2^Department of Pathology, University of California, San Diego, CA, United States; ^3^California Institute for Regenerative Medicine, California State University San Marcos, San Marcos, CA, United States; ^4^Department of Neurological Surgery, University of Washington, Seattle, WA, United States

**Keywords:** unipolar brush cells, Tbr2, cerebellum, development, cell migration

## Abstract

Previous studies demonstrated specific expression of transcription factor Tbr2 in unipolar brush cells (UBCs) of the cerebellum during development and adulthood. To further study UBCs and the role of Tbr2 in their development we examined UBC morphology in transgenic mouse lines (reporter and lineage tracer) and also examined the effects of Tbr2 deficiency in *Tbr2* (MGI: *Eomes*) conditional knock-out (cKO) mice. In *Tbr2* reporter and lineage tracer cerebellum, UBCs exhibited more complex morphologies than previously reported including multiple dendrites, bifurcating dendrites, and up to four dendritic brushes. We propose that “dendritic brush cells” (DBCs) may be a more apt nomenclature. In *Tbr2* cKO cerebellum, mature UBCs were completely absent. Migration of UBC precursors from rhombic lip to cerebellar cortex and other nuclei was impaired in *Tbr2* cKO mice. Our results indicate that UBC migration and differentiation are sensitive to Tbr2 deficiency. To investigate whether UBCs develop similarly in humans as in rodents, we studied Tbr2 expression in mid-gestational human cerebellum. Remarkably, Tbr2^+^ UBC precursors migrate along the same pathways in humans as in rodent cerebellum and disperse to create the same “fountain-like” appearance characteristic of UBCs exiting the rhombic lip.

## Introduction

Unipolar brush cells (UBCs) are a type of glutamatergic interneuron localized to the granule cell layer of the cerebellum and are especially abundant in the posterior vermis (Floris et al., 1994; Dino et al., 1999; Nunzi et al., 2001; Kalinichenko and Okhotin, 2005; Mugnaini et al., 2011). Besides the cerebellum, UBCs are also found in the dorsal cochlear nucleus (Borges-Merjane and Trussell, 2015). UBCs possess a unique morphology classically described with a single short dendrite ending in a brush-like spray of dendrioles (Floris et al., 1994; Mugnaini and Floris, 1994; Dino et al., 1999; Morin et al., 2001; Nunzi et al., 2001; Kalinichenko and Okhotin, 2005; Millen and Gleeson, 2008). These dendrioles interact with a single mossy fiber rosette that, upon white matter stimulation, results in a prolonged excitatory event and constitutes a link in the mossy fiber-granular cell-Purkinje pathway (Rossi et al., 1995; Dino et al., 1999).

Two subtypes of UBCs have been characterized by marker expression and neurochemical phenotype. Type I UBCs express calretinin (Arai et al., 1991; Floris et al., 1994; Morin et al., 2001; Nunzi et al., 2002; Kalinichenko and Okhotin, 2005; Englund et al., 2006) and type II UBCs express mGluR1α (Takacs et al., 1999; Spatz, 2001; Kalinichenko and Okhotin, 2005). Previous studies suggest that Tbr2 (MGI: *Eomes*) expression in UBCs continues from development to adulthood and Tbr2 serves as a pan-UBC marker (Englund et al., 2006; Pimeisl et al., 2013). Tbr2^+^ UBCs are especially abundant in the internal granular layer of the nodulus (lobule X) and ventral uvula (lobule IX) with additional accumulations in lobules VI-VII (Englund et al., 2006).

In the embryonic cerebellum there are distinct progenitor compartments that produce GABAergic and glutamatergic neurons. The former originate from the ventricular zone (VZ) of the fourth ventricle, while the latter are generated from the rhombic lip (Englund et al., 2006; Fink et al., 2006; Millen and Gleeson, 2008). Among the glutamatergic neurons, UBCs are born from the rhombic lip between E14.5 and E19.5 in mouse. During pre- and postnatal differentiation UBC precursors sequentially express Pax6 and then Tbr2 (Sekerkova et al., 2004; Englund et al., 2006). UBCs migrate from the rhombic lip through developing white matter to cerebellar cortex and to the dorsal cochlear nucleus producing a “fountain-like” migration in sagittal histological sections (Englund et al., 2006).

Tbr2 is a specific marker of glutamatergic neurons and progenitor cells in several regions of the mammalian central nervous system (Hevner et al., 2006) and is essential for brain development (Mihalas and Hevner, 2017). In the developing cerebral cortex, Tbr2 is essential for the differentiation of intermediate progenitor cells to retain frontal identity and advance neuronal differentiation. In *Tbr2* cKO mice the development of neocortex and dentate gyrus are impaired (Arnold et al., 2008; Hodge et al., 2012; Elsen et al., 2013; Mihalas et al., 2016). However, the effects of *Tbr2* cKO on the cerebellum have not been characterized.

To determine if Tbr2 is necessary for the development of UBCs we examined *Tbr2* cKO mice. We found that UBCs are absent from the *Tbr2* cKO cerebellum as determined by immunohistochemistry (IHC) for markers of all UBC subtypes. We also investigated the potential role of Tbr2 in human cerebellum development by studying Tbr2 expression patterns in human fetal cerebella (19–20 weeks of gestation). We found that Tbr2^+^ cells were localized in migration pathways similar to those in mice (Englund et al., 2006). Intriguingly, the use of reporter lines for Tbr2 expression revealed an unprecedented level of morphological detail for individual UBCs in the rodent. This includes novel UBC morphologies with multiple brushes suggesting that UBCs are more complex than previously thought.

### Experimental Procedures

#### Animals and Tissue Preparation

All animals were used in accordance with a protocol approved by the Institutional Animal Care and Use Committee and the Seattle Children’s Research Institute. *Tbr2*^*flox/flox*^ mice (Intlekofer et al., 2008) were crossed with *Nes11*^*Cre*^ mice (Tronche et al., 1999) and *Tbr2*^*lacZ*^ mice (Russ et al., 2000). Additionally, *Eomes*^*CreER*^ mice were crossed with *Ai14*-reporter mice as previously published (Pimeisl et al., 2013) and *Tbr2-GFP*-knockin mice (Arnold et al., 2009) were studied as well. All mice were maintained on a C57BL/6;ICR mixed background. Plug date was defined as embryonic day (E) 0.5. Embryos were immersion fixed in 4% phosphate-buffered paraformaldehyde (PFA) for 4-12 hrs, neonatal pups were perfused under cryoanesthesia, and older pups and adult mice were perfused under isoflurane anesthesia. Mice were perfused with PBS followed by cold 4% PFA. Brains were removed and post-fixed for 16–20 h at 4°C, cryprotected with increasing concentrations of sucrose (10, 20, 30%), and embedded in OCT. Sections were cut at 12 μm and stored at −80°C prior to immunostaining.

#### Tamoxifen Induced Recombination

To activate CreER, 6 mg of tamoxifen (Sigma, prepared as a 20 mg/ml stock solution in corn oil) per 35 g of body weight was administered by intraperitoneal injection (i.p.) to pregnant mice at E14.5.

#### Bromodeoxyuridine (BrdU) Administration

Bromodeoxyuridine (Sigma) was dissolved in 0.1M phosphate-buffered saline pH 7.4 (PBS) and 50 mg/g of body weight of BrdU were given to pregnant mice 45 min prior to sacrifice by a single i.p. injection.

#### Immunohistochemistry

Primary mouse monoclonal antibodies included: anti-calretinin (1:1000) from Chemicon, anti-PCNA (1:2000) from Chemicon, and anti-PLCB 4 (1:400) from Santa Cruz. The rat monoclonal anti-BrdU (1:400) from Harlan Sera-Lab (Loughborough, United Kingdom) was also used. Rabbit polyclonal antibodies included: anti-calretinin (1:500) from Swant, anti-Tbr1 (1:1000) from ProSci, anti-mGluR1α (1:500) from Novus Biologicals, anti-Ki67 (1:100) from DAKO, and anti-Tbr2 (1:2000) from RH’s laboratory (Englund et al., 2006). Secondary antibodies, Alexa-488 and Alexa-594, against mouse, rat, and rabbit (1:400) were obtained from Invitrogen. For antigen enhancement, cryostat sections were boiled and cooled twice in 10 mM sodium citrate and rinsed in PBS. Sections that had been labeled with BrdU were treated with 2N HCL for 30 min at 37°C and then rinsed in PBS. Following pretreatments, tissue was incubated with blocking solution for 30 min and then incubated with primary antibodies overnight at 4°C. Sections were rinsed and incubated in appropriate secondary antibodies for 2 h at room temperature, counterstained for 10 min with filtered 1% DAPI (Sigma) in PBS, and cover-slipped.

#### Human Cerebellum Tissue

All human cerebellum tissue was used in accordance with Seattle Children’s Research Institute standard operating protocols for obtaining donated tissue from autopsies. This includes rigorous standards for obtaining consent from subject(s) and/or their legal guardians/representatives and de-identification measures to protect patient identity and privacy. Use of this tissue was approved by the Institutional Review Board (IRB) of Seattle Children’s Hospital.

Autopsy specimens from the cerebella of 19- and 20-week post-gestational fetuses were fixed for 2 weeks in formalin, embedded in paraffin (FFPE), and sectioned at 7 microns. Human tissue sections were prepared using an automated stainer (Roch/Ventana) and deparaffinized with heat. For IHC, we used primary monoclonal anti-Tbr2 (1:100, ThermoFisher) and secondary rabbit anti-mouse Alexa-Fluor 488 (1:100, Invitrogen) as directed per the automated stainer manufacturer instructions. Sections were then cover slipped and preserved with Permount. For DAB, antigen retrieval was performed at 100°C in a Tris-EDTA based solution (pH9) and blocked for endogenous peroxidase with H_2_0_2_ prior to incubation with anti-Tbr2 (1:100, ThermoFisher). We used a rabbit anti-mouse HRP (Roche Tissue Diagnostics) as a secondary antibody per kit instructions. TSA amplification was performed before incubation with tertiary antibody (rabbit anti-HW HRP; Roche Tissue Diagnostics) per kit instructions. DAB was developed followed by hematoxylin as counterstain. Sections were then cover slipped and preserved with Permount.

#### X-gal Histochemistry

For X-gal staining of tissue sections, brains were fixed with 4% PFA in PBS at 4°C for 60 min, washed in PBS, equilibrated in 20% sucrose prior to mounting in OCT compound and frozen at −80°C. Frozen sections (16 μm thick) were cut by cryostat. Tissue was fixed in 2% formaldehyde and 0.2% gluteraldehyde in PBS solution for 60 min at 4°C, rinsed twice in 2 mM Cl_2_, 0.01% sodium deoxycholate, 0.02%NP-40 (in 0.1 M phosphate-buffered saline pH7.4), and then stained at 37°C for 8 h in X-gal stain buffer [2 mg X-gal in 2 mM MgCl2, 0.01% sodium deoxycholate, 0.02% NP-40, 5 mM K3Fe(CN)6, 5 mM K4F(CN)6 in 0.1M phosphate buffer].

#### Microscopy and Image Processing

Epifluorescence and confocal imaging were performed on a Zeiss LSM70 confocal microscope as previously described (Kahoud et al., 2014). Human samples and IHC hybridized tissue, were analyzed by conventional light microscopy on Zeiss confocal microscope (Zeiss).

## Results

### Unipolar Brush Cells Exhibit Complex Morphology

**FIGURE 1:**
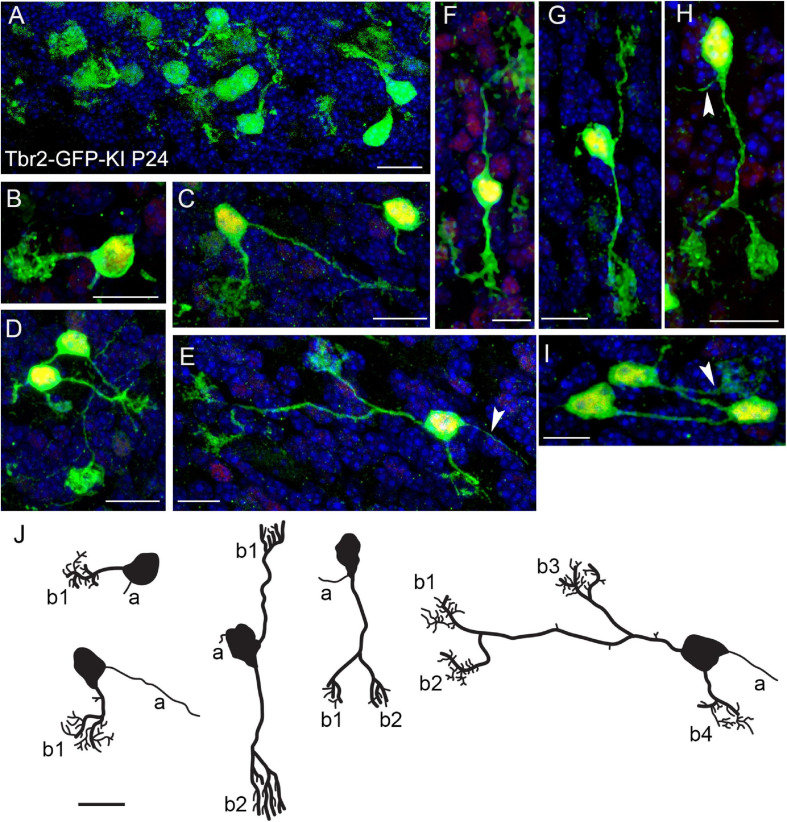
Morphology of Tbr2-GFP^+^ cells in the adult cerebellum is more complex than previously characterized. **(A)** Tbr2-GFP^+^ cells in the P24 nodulus. **(B-D)** Several UBCs exhibit classic morphology consisting of an axon and a single dendrite terminating in a brush-like spray of dendrioles. The *Tbr2-GFP*-KI transgenic mouse enables complex morphological features of the UBCs to be visualized at a higher resolution than previously described. **(E)** A UBC possessing a long dendrite that bifurcates twice, resulting in a cell with three brushes from one dendrite and a single brush from a separate dendrite. An axon is also visible to the right of the cell body (arrow). **(F)** A UBC that appears bipolar in the orientation of its dendrites both of which terminate in brushes. **(G,H)** UBCs possessing two brushes, one from separate dendrites **(G)** and another from a bifurcated dendrite **(H)**, which also has a visible axon (arrow). **(I)** A cluster of UBCs, one of which has a visible brush in the field of view. **(J)** Tracings of UBCs illustrating diverse morphologies. Axons (a) and brushes (b, numbered) are indicated. Immunolabeling in **(A–I)**: Tbr2 in red, Tbr2-GFP in green, DAPI in blue. Note low levels of Tbr2 immunostaining of some of the cerebellar granule neurons compared with greater intensity in UBCs. Scale bars: 10 μm.

Previous studies in our lab indicate that Tbr2 is constitutively expressed in all UBCs (Englund et al., 2006; Pimeisl et al., 2013). To study UBC morphology we took advantage of this finding by examining the cerebella of *Tbr2-GFP* knock-in (KI) transgenic mice (Arnold et al., 2009). In the P24 cerebellum we found a multitude of GFP^+^ UBCs that were especially abundant in the nodulus (Figure 1A). Among the cells we observed in the *Tbr2-GFP*-KI cerebellum we found a number of UBCs exhibiting “classic” single dendritic brush UBC morphology (Figures 1B–D). (Historically, the “unipolar” descriptor of UBCs has referred to the number of dendrites.) However, we also found that the Tbr2-GFP construct provides enhanced visualization of cellular morphology including more complex morphologies than previously described. For example, some UBCs possessed two dendrites and an axon with one of the dendrites bifurcating twice to result in a cell with four brushes (Figure 1E). We also observed “bipolar” UBCs that extended two main dendrites in opposite directions (Figures 1F,G), as well as “unipolar” UBCs with multiple brushes that extended from a single bifurcated main dendrite (Figure 1H). Reflecting their abundance in the nodulus, UBCs were sometimes localized in clusters with overlapping processes (Figure 1I).

**FIGURE 2:**
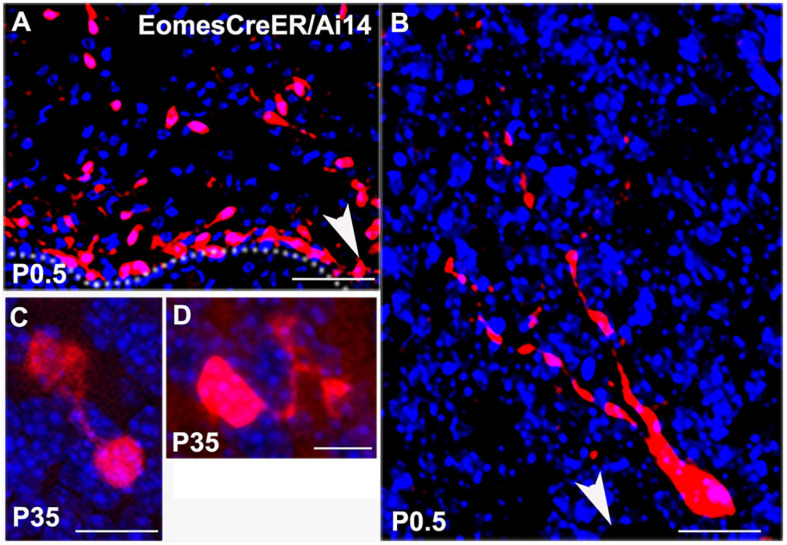
Lineage tracing of Tbr2^+^ cells in the cerebellum using *Eomes^*CreER*^/Ai14*-reporter mice that were injected with tamoxifen at E14.5 and sacrificed at time points indicated in panels. **(A)** Tbr2^+^ cells migrating from the rhombic lip at P0.5. Arrow indicates direction of the rhombic lip. **(B)** Migrating Tbr2^+^ UBC progenitor with visible leading processes. Arrow indicates direction of the rhombic lip. **(C)** Tbr2^+^ UBC with a singular brush in the P35 vestibulocerebellum. **(D)** Tbr2^+^ UBC with a double brush in the P35 vestibulocerebellum. Scale bars: 50 μm in **(A)**, 10 μm in **(B–D)**. Immunostaining: Ai14 in red and DAPI in blue.

These data were corroborated by UBC morphologies in *Eomes^*CreER*^/Ai14* reporter mice. When we injected tamoxifen at E14.5 for lineage tracing of Tbr2^+^ UBCs from precursor stages we were able to visualize UBC precursors migrating from the rhombic lip at P0.5 (Figures 2A,B). At later postnatal stages the UBC soma and dendrites filled with RFP and showed singular brush morphology (Figure 2C) and double brush morphology (Figure 2D). Since RFP labeling only reflected *Eomes*-expressing cells that underwent *Ai14* recombination at the time of tamoxifen administration, UBCs were only sparsely labeled in these experiments.

Due to the clustering of UBCs labeled using either transgenic line (Figures 1A, 2A), we are unable to determine an accurate proportional count of multiple brush and/or dendrite UBCs in either transgenic line. Within these dense clusters we cannot clearly determine which brush(es) and dendrite(s) belong to which soma to obtain accurate counts of single vs. multi-brush UBCs. Even the *Ai14* reporter mice, which label only a subset of UBCs based on recombination efficiency and day of tamoxifen administration, produced clustering that prevented accurate counting. However, Figures 1, 2 depict examples of UBCs with single and multiple brushes from low density regions where we can reliably attribute the observed dendrite(s) and brush(es) to a specific soma. Repeated observations in several animals of both transgenic lines suggest these are not rare cells. However, the fraction of UBCs with multiple brushes could not be determined, considering the technical limitations due to dense clustering in several regions of the cerebellum.

**FIGURE 3:**
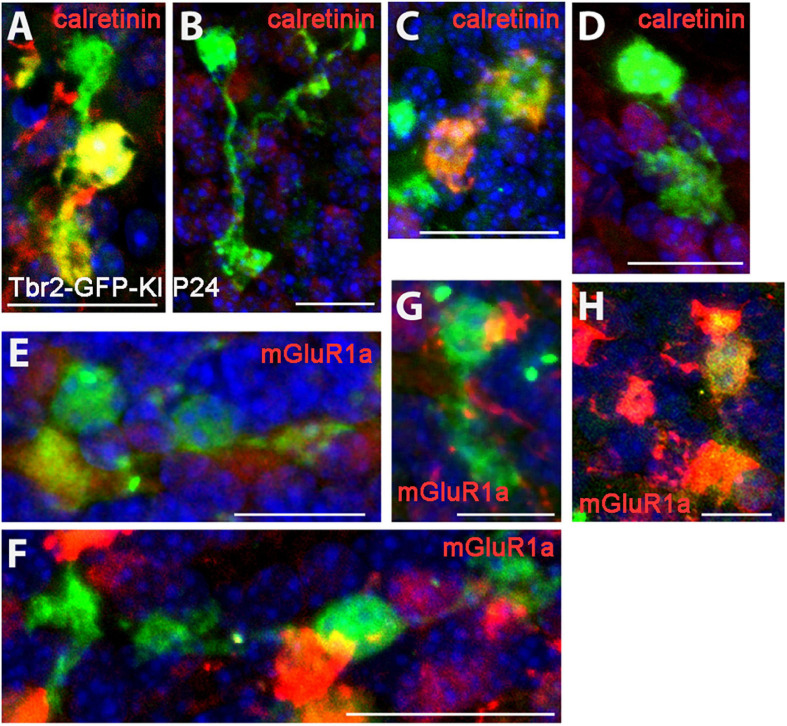
Expression of type I and type II UBC markers in Tbr2-GFP^+^ cells in the P24 nodulus. **(A–D)** Calretinin (red) immunostaining in Tbr2-GFP^+^ cells (green) is present in UBCs with multiple brushes **(A)** as well as absent in UBCs with multiple brushes **(B)**. Similarly, single brush UBCs may be calretinin^+^ **(C)** or calretinin^–^ **(D)**. **(E–H)** mGluR1α (red) expression in Tbr2-GFP^+^ cells is present in UBCs with multiple brushes **(E)** as well as absent in UBCs with multiple brushes **(F)** and single brush UBCs may be either mGluR1α– **(G)** or mGluR1α+ **(H)**. Scale bars: 15 μm **(A)**, 20 μm **(B)**, 15 μm **(C)**, 20 μm **(D–F)**, and 10 μm **(G,H)**.

When we examined the expression of type I (calretinin^+^) and type II (mGluR1α^+^) UBC markers in the Tbr2-GFP^+^ cells we found no morphological differences between the two subtypes. Both markers were present in single-brushed UBCs and double-brushed UBCs (Figure 3). Interestingly, in some examples only one brush of a double-brushed UBC was positive for a specific marker such as calretinin (Figure 3A). Similarly to above, examples of cells in which clear attribution of brushes and/or dendrites to a single soma are provided.

### Cerebellar Development and Organization in the Tbr2 cKO Brain

**FIGURE 4:**
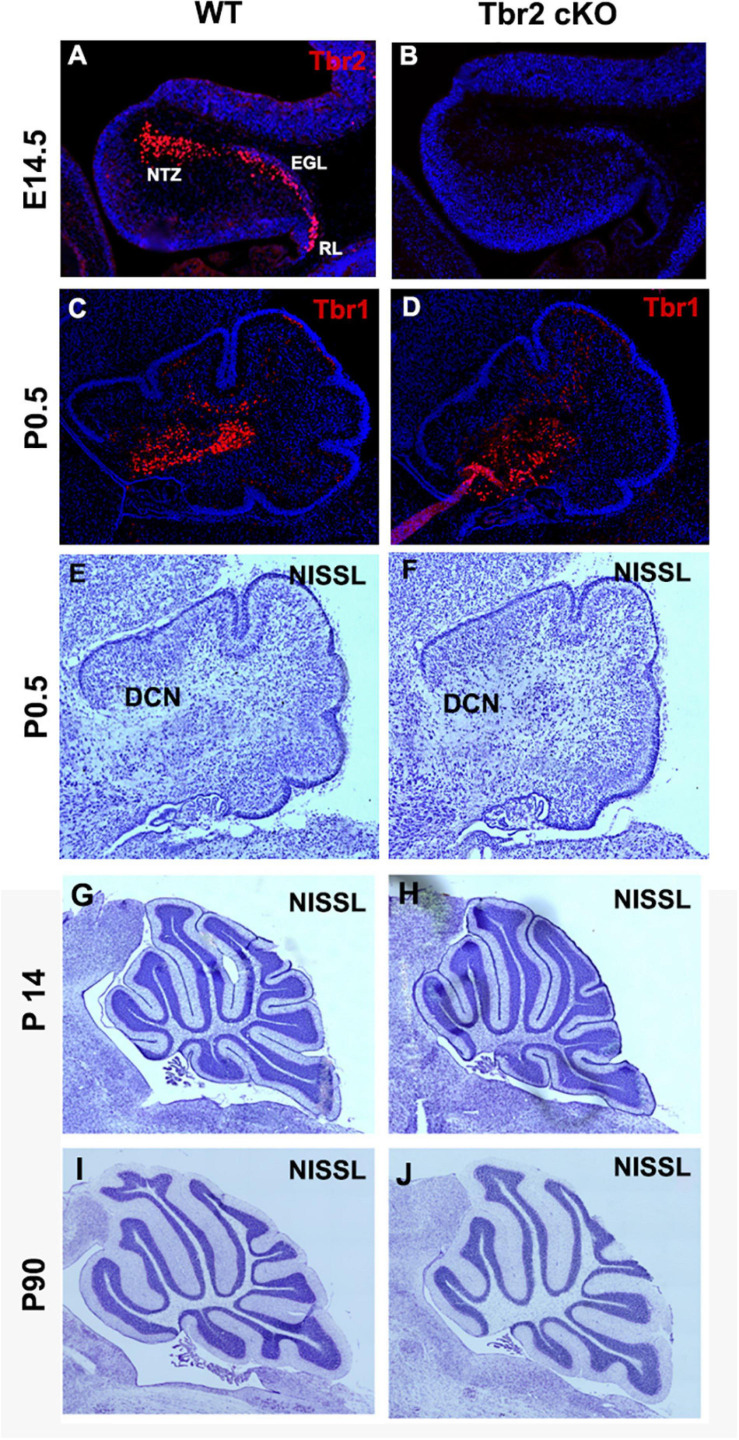
Ablation of Tbr2 at E11 does not appear to affect other cell populations and results in a nearly normal cerebellar morphology. **(A)** A migratory stream of Tbr2^+^ progenitors to the nuclear transitory zone, site of the future deep cerebellar nuclei, in the developing cerebellum is visible at E14.5 in the WT cerebellum, **(B)** while these cells are not Tbr2^+^ in the cKO, however, morphology of the cerebellum appears similar to the WT cerebellum. **(C,D)** The deep cerebellar nuclei neurons are not affected in the *Tbr2* cKO cerebellum, however, effects of Tbr2 inactivation in this cell population may be masked due to the developmental timing of DCN neurogenesis. **(E,G,I)** Nissl staining of the wild-type cerebellum and **(F,H,J)** *Tbr2* cKO cerebellum at multiple timepoints demonstrates that overall morphology appears very similar to WT cerebella at matched time points and medial sections.

To determine the importance of *Tbr2* in cerebellar development, we crossed *Tbr2*^*flox/flox*^ mice (Intlekofer et al., 2008) with *Nes11*^*Cre*^ mice to generate *Tbr2* conditional knockouts (cKO). The *Nes11*^*Cre*^ mouse expresses Cre recombinase throughout stem/progenitor cells of the developing central nervous system, including the cerebellum, beginning on E11 (Tronche et al., 1999). As expected, no Tbr2 immunoreactivity was detected in the *Tbr2* cKO cerebellum (Figures 4A,B). We previously reported that glutamatergic projection neurons of the deep cerebellar nuclei (DCN) are derived from the rhombic lip and generated from E10.5 to E12.5 in mice (Miale and Sidman, 1961; Fink et al., 2006). As these cells migrate from the rhombic lip to the nuclear transitory zone (NTZ) they sequentially express Pax6, Tbr2, and Tbr1 (Fink et al., 2006). Accordingly, we examined the glutamatergic DCN neurons to see if they were abnormal in *Tbr2* cKO mice due to inactivation of Tbr2 mid-neurogenesis. Tbr1 IHC suggests that absence of Tbr2 starting at E11 does not substantially affect the population of Tbr1^+^ DCN projection neurons observed in early postnatal mice (Figures 4C,D). However, some DCN projection neurons were presumably generated before *Tbr2* inactivation so DCN phenotypes in the *Tbr2* cKO may be masked. Furthermore, while examining overall cerebellar morphology at multiple stages we noticed no consistent deformities or disorganization in the *Tbr2* cKO cerebellum compared to the WT (Figures 4C–J).

**FIGURE 5:**
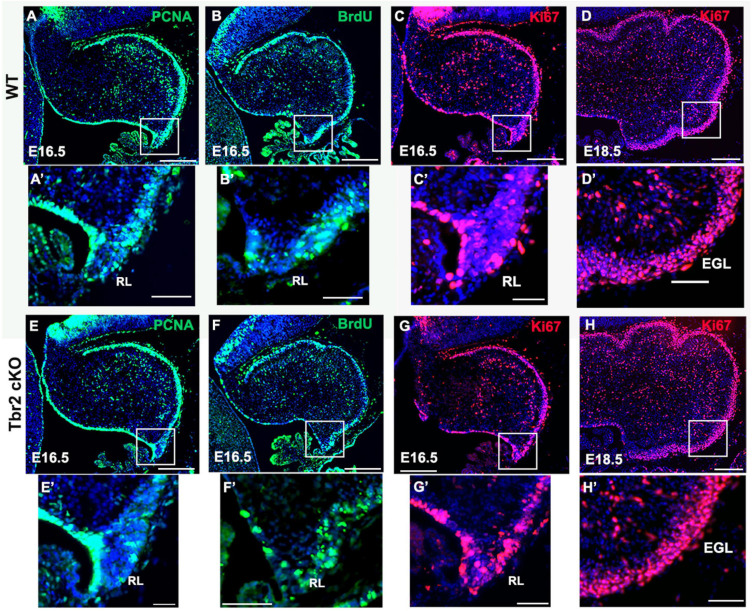
Proliferation in the cerebellum and rhombic lip does not appear to be impaired by *Tbr2* ablation. WT cerebellum **(A)** and *Tbr2* cKO cerebellum **(E)** stained with the proliferative marker PCNA shows no apparent difference in proliferation. Following a 30-min pulse of BrdU prior to sacrifice, the WT **(B)** and cKO **(F)** cerebella show no significant difference in BrdU immunolabeling within the cerebellum or the rhombic lip (**B’**,**F’**, respectively). Furthermore, Ki67 labeling on E 16.5 and E 18.5 indicate no variations between WT **(C,D)** or cKO **(G,H)** cerebella, within the rhombic lip at E16.5 **(C’,G’)**, or external germinal layer (EGL) at 18.6 **(D’,H’)**. This suggests the *Tbr2* cKO does not result in an impairment of proliferation during the period of peak UBC genesis. Scale bars: **(A–F)**: 100 μm; **(A’–F’)**: 50 μm.

To determine if the absence of Tbr2 altered proliferation in the rhombic lip and other cerebellar germinal zones we studied proliferation markers PCNA and Ki67 in E16.5 control and *Tbr2* cKO cerebellum (Figure 5). We found no apparent difference in proliferation between WT and *Tbr2* cKO cerebella. Similarly, acute BrdU labeling of E16.5 cerebella revealed no defects in cell proliferation, neither in the rhombic lip where the UBC progenitor niche is located (Englund et al., 2006) nor elsewhere in the developing cerebellum (Figures 5B,F). Our data suggest that ablation of *Tbr2* does not noticeably impact overall proliferation within the developing cerebellum during the peak of UBC genesis.

### Multiple UBC Subtypes Are Absent From Tbr2 cKO Cerebellum

**FIGURE 6:**
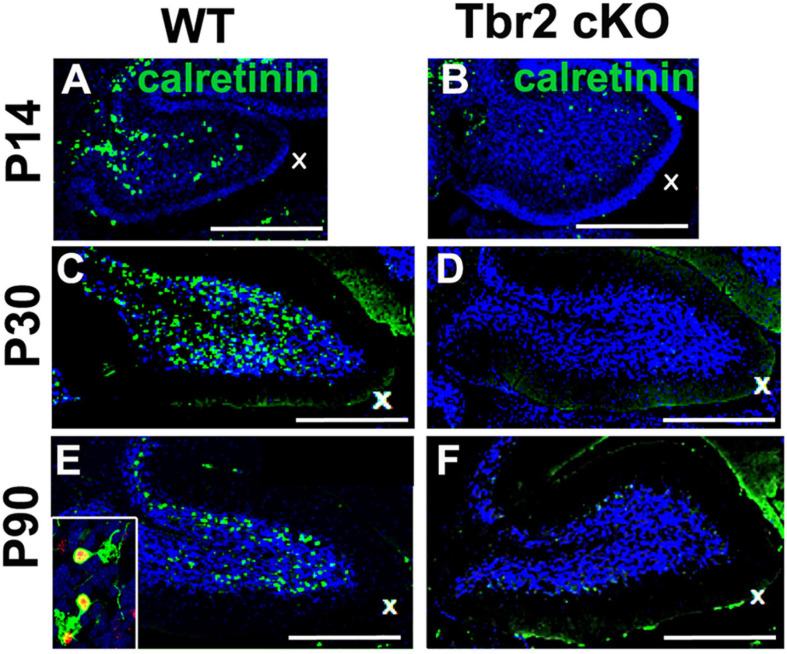
Tbr2 is required for the differentiation of calretinin^+^ unipolar brush cells. **(A,C,E)** Calretinin^+^ cells are present in the adult WT cerebellum but missing in the adult *Tbr2* cKO cerebellum **(B,D,F)** at all time points observed suggesting a defect of UBC genesis, differentiation, or migration. In panel **(E)** the inset shows a close up of Tbr2^+^ (red)/calretinin^+^ (green) UBCs found in the WT cerebellum, a cell type that is missing in the *Tbr2* cKO cerebellum. Scale bars: 500 μm in **(A,B)**, 200 μm **(C–F)**.

Because our previous studies demonstrated Tbr2 expression in multiple UBC subtypes, we examined the *Tbr2* cKO cerebellum for markers of type I and type II UBCs. Immunoreactivity for calretinin, a type I UBC marker, was markedly reduced in *Tbr2* cKO cerebellum as compared to controls (Figure 6). No strongly calretinin^+^ cells were present in the adult *Tbr2* cKO cerebellar cortex, not even in the granular layer of lobules IX and X where UBCs are normally abundant (Figures 6D,F). This suggests that differentiation or survival of calretinin^+^ UBCs was severely reduced or absent and not merely delayed. It is worth noting that some calretinin immunoreactivity was detected in the mutant cerebellum; however, multiple cerebellar cell types express calretinin at low levels, including granule cells, subsets of mossy and climbing fibers, and Lugaro cells (Bearzatto et al., 2006). We did not observe UBC phenotypes such as dendritic brush morphology in the remaining weakly calretinin^+^ cells.

**FIGURE 7:**
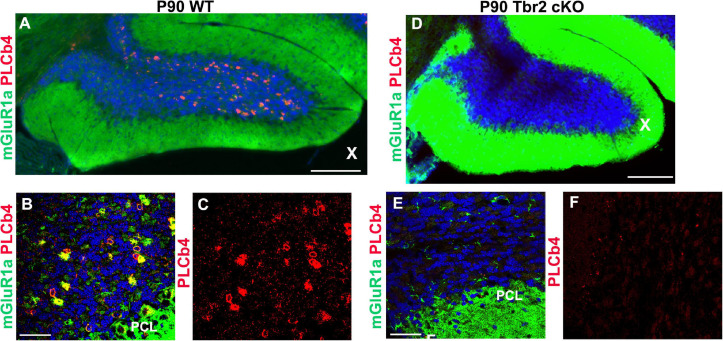
Tbr2 is required for the specification of the mGluR1α/PLCβ4 UBC subtype. **(A–C)** Type II UBCs express mGluR1α and PLCβ4 in the WT cerebellum. **(D–F)** These cells are absent in the *Tbr2* cKO cerebellum. **(C)** PLCβ4 immunostaining shows a multitude of these cells in the WT cerebellum, but none in the *Tbr2* cKO **(F**). Scale bars: **(A,D)**: 200 μm; **(B,C,E,F)**: 50 μm.

To determine if type II UBCs are affected by *Tbr2* ablation, cells that express mGluR1α and PLCβ4 were also studied (Nunzi et al., 2002; Englund et al., 2006). An abundance of mGluR1α^+^ and PLCβ4^+^ cells were detected in the granular layer of the WT cerebellum (Figures 7A–C) but these cells were absent from the granular layer of the *Tbr2* cKO cerebellum (Figures 7D–F). Based on these findings we conclude that inactivation of *Tbr2* during UBC development results in a lack of cells expressing UBC markers in the internal granular layer of the cerebellum.

### *Tbr2* Inactivation Disrupts Migration of Unipolar Brush Cell Progenitors

**FIGURE 8:**
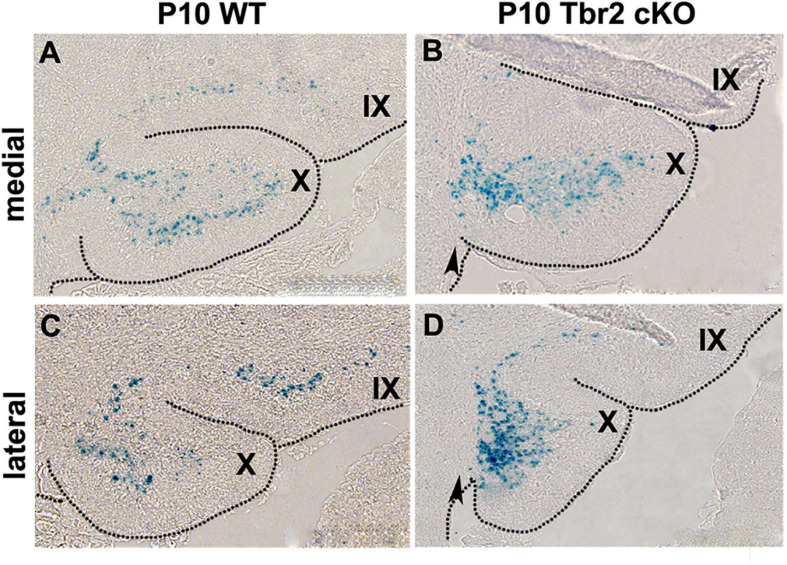
β-gal histochemistry in *Tbr2*^*LacZ/flox*^ cerebellum enables the tracing of putative UBC progenitors in the *Tbr2* cKO cerebellum. **(A,C)** In the WT cerebellum the Tbr2-expressing (β-gal^+^) UBCs are in the granular layer of lobules X and IX in the medial and lateral cerebellum. **(B,D)** In the *Tbr2* cKO cerebellum most β-gal^+^ cells accumulated in posterior lobule X, adjacent to the site of the former rhombic lip (arrow) suggesting a migration defect.

To determine the fates of defective UBC precursors in *Tbr2* cKO cerebellum, the *Tbr2*^*lacZ*^ reporter allele was used. β-galactosidase (*lacZ* gene product) activity was compared in *Tbr2* cKO (*Nes11^*Cre*^;Tbr2^*lacZ/flox*^*) and heterozygous control (*Nes11*^*Cre*^;*Tbr2*^*lacZ/+*^) mice. The overall number of β-galactosidase^+^ cells in anatomically comparable parasagittal sections of P10 cerebella appeared similar or slightly increased in Tbr2 cKO as compared to WT cerebellum. However, the localization of β-galactosidase^+^ cells in the *Tbr2* cKO cerebellum was abnormal. Instead of spreading throughout the granular layer of lobules IX and X as in WT cerebellum (Figures 8A,C) β-galactosidase^+^ cells in *Tbr2* cKO cerebellum were clustered in developing white matter near the site of the former rhombic lip, suggesting a defect of UBC precursor migration (Figures 8B,D). However, as shown in Figures 6, 7, we did not see expression of UBC markers such as calretinin and mGluR1α in these areas of the cerebellum, nor did we see these markers expressed in the granular layer of the adult mouse (P90). Thus, we conclude that there is an early migratory defect present at P10 in the cKO cerebellum and adult cerebella fail to express markers of mature UBCs.

**FIGURE 9:**
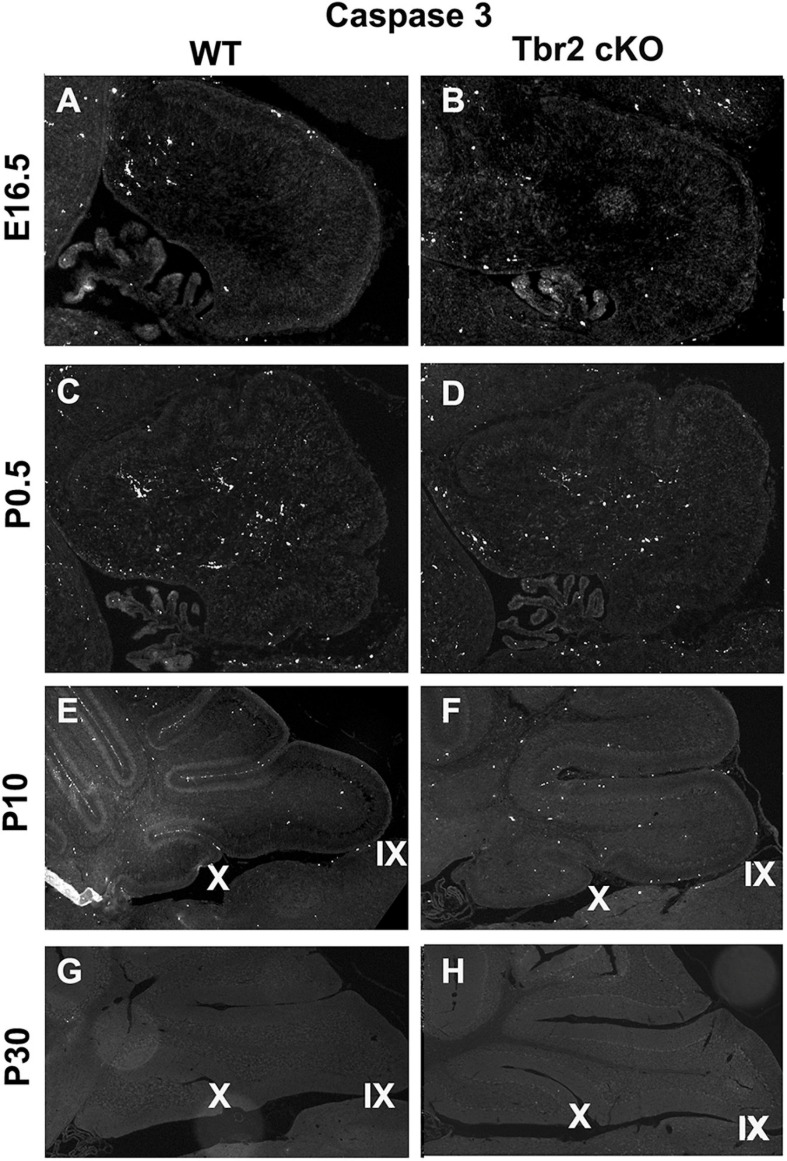
Comparison of activated caspase3 (AC3) immunoreactivity in the WT and *Tbr2* cKO cerebella does not indicate that UBC precursors undergo apoptosis in the nodulus of the mutant cerebellum. **(A,C)** The WT cerebellum shows little AC3 activity at early stages of postnatal development and the *Tbr2* cKO cerebellum shows similar levels and localizations of caspase3 activity **(B,D)**. **(E)** At P10 there is increased immunoreactivity in the WT cerebellum particularly along pial surfaces. **(F)** In the *Tbr2* cKO cerebellum there is increased immunoreactivity for caspase3 compared to earlier stages predominantly in the white matter and molecular layers of the cerebellum. The pattern of AC3 immunostaining in the mutant cerebellum does not suggest that putative UBC progenitors that failed to migrate out of the rhombic lip are undergoing apoptosis. During adulthood in the mouse there is no apparent AC3 in either the WT **(G)** or mutant **(H)** cerebellum.

Since proliferative activity did not appear to differ between WT and *Tbr2* cKO cerebella (Figure 4) we investigated cell death in the *Tbr2* cKO using activated caspase-3 (AC3) as a marker of apoptotic cells. During early stages of cerebellar development (E16.5 and P0.5) no differences of AC3 expression were detected between WT and *Tbr2* cKO cerebella (Figures 9A–D). Differences in the pattern of AC3 expression were detected during postnatal development. In P10 WT cerebellum, AC3^+^ cells were localized mainly below the pial surface (Figure 9E). In contrast, AC3^+^ cells were scattered in the white matter and molecular layer of *Tbr2* cKO cerebellum (Figure 9F). The significance of this expression pattern is unclear; there is no abundance of AC3^+^ cells in the Tbr2 cKO either at the site of the rhombic lip, where β-gal^+^ cells were observed at P10 (Figures 8B,D), or within the granular layer. Nor are there differences in the expression pattern of AC3^+^ cells at P30 between WT and cKO cerebella (Figures 9G,H).

### Tbr2^+^ Cells Originate From the Rhombic Lip of the Human Fetal Cerebellum

**FIGURE 10:**
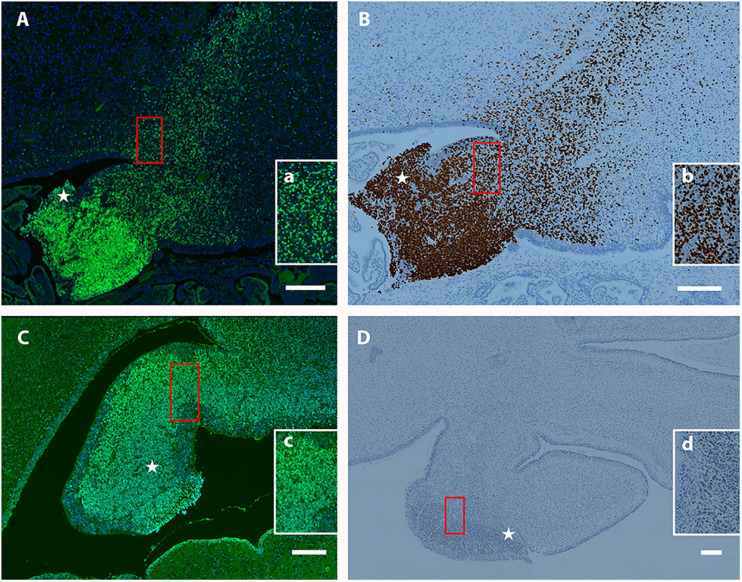
Localization of Tbr2^+^ UBCs in the developing human cerebellum. **(A–D)** Sagittal sections; Top: Superior, Bottom: Inferior, Left: Anterior, Right: Posterior. Star (⋆) indicates rhombic lip. **(A)** At 19 weeks a migratory stream of Tbr2^+^ UBCs (green) and DAPI (blue) from the rhombic lip is visible with accumulations in the nodulus and uvula. The boxed (red) region is depicted at higher magnification in **(a)**. **(B)** At 19 weeks the same fountain of Tbr2^+^ UBCs from rhombic lip is confirmed by a different immunohistochemical method (brown-DAB). Boxed (red) region shown at higher magnification in **(b)**. **(C)** At 20 weeks, Tbr2^+^ UBCs (green) are shown near the rhombic lip shown by immunofluorescence with boxed (red) region shown at higher magnification in **(c)**. **(D)** At 20 weeks Tbr2^+^ UBCs (brown-DAB) are shown near the rhombic lip in greater detail in **(d)**. Scale bar: 200 μum in **(A–D)**.

Previously, we observed that Tbr2^+^ UBC precursors are abundant in the rhombic lip and display a “fountain-like” appearance emanating from this proliferative zone in the embryonic mouse (Englund et al., 2006). In our characterization of the Tbr2 cKO cerebella we also observed this in our WT controls (Figure 4A). To determine if UBC precursors also arise from the rhombic lip in humans during early developmental periods and also express Tbr2, the cerebella of 19- and 20-week post-conceptional gestation fetuses were stained with antibodies against Tbr2. At 19 post-conceptional weeks, the Tbr2^+^ UBC precursors were abundant in the rhombic lip and in migration streams emanating from it (Figures 10A,B) that display a “fountain-like” appearance nearly identical to the migratory streams observed in the developing mouse cerebellum (Figure 4A; Englund et al., 2006). At 20 post-conceptional weeks, the Tbr2^+^ UBC precursors had a similar distribution and appeared to enter the internal granular layer of nodulus (lobule X) and uvula (lobule IX) (Figures 10C,D). Based on these data, we conclude that there are striking early developmental similarities in UBC origin and migration in human and mouse.

## Discussion

### Unipolar Brush Cells Exhibit Diverse and Complex Morphologies

Utilizing GFP knock-in and *Eomes^*CreER*^/Ai14* lineage tracer mice for confocal microscopy, we have generated high resolution images of UBC morphology and demonstrated that these cells have more complex morphology than previously reported. The *Tbr2-GFP* KI mice have the advantage of labeling all cells that are actively expressing Tbr2 at the time of analysis; since Tbr2 expression is maintained by UBCs in adult animals, this includes all UBCs. However, this reporter provides little data on cell lineage, birth dating, or definitive cellular identity absent confirmatory co-labeling and/or morphological analysis. Using this line, we were able to demonstrate complex morphology of the UBCs due to the cytosolic spread of the GFP fluorophore and confirmed their identity on the basis of IHC and distinctive morphological features. Conversely, the *Eomes^*CreER*^/Ai14* lineage tracer provides precise birth dating and cell lineage tracing based on the time of tamoxifen administration. However, the recombination efficiency of these lines is often low and due to its birth dating capabilities, the labeled cells represent a fraction of a given cell population. Despite the lower relative numbers of cells labeled in the *Eomes^*CreER*^/Ai14* reporter mice, we observed similar multiple brush morphologies. Thus, our two transgenic lines provided complementary data on the morphology of UBCs, the identity of fluorophore-positive cells, and the timing of UBC genesis.

Previous literature describes UBCs as having a single dendritic process, either stubby or slender in shape, terminating with a compact arrangement of short dendrioles forming a brush-like structure and a single axon emerging from the cell body (Floris et al., 1994; Mugnaini and Floris, 1994; Dino et al., 1999; Morin et al., 2001; Nunzi et al., 2001; Kalinichenko and Okhotin, 2005; Millen and Gleeson, 2008; Mugnaini et al., 2011). Kalinichenko and Okhotin (2005) described three UBC morphological categories on the basis of the size and shapes of the brush noting some with very short dendritic stems (type 1), some with a dendrite 20–30 μm long that may divide to give rise to two separate brushes (type 2), and type 3 neurons with a dendrite up to 50 μm crowned by a short brush. Using this morphological nomenclature, we observed some type 2 UBCs that had a bifurcating dendrite but we also found several UBCs that possessed multiple dendrites and more than two brushes, albeit at a low frequency of occurrence. Using a neurochemical basis of UBC categorization, we found no relationship between a multiple brush phenotype and expression of calretinin or mGluR1α. The name “unipolar” brush cell may be outmoded in light of our findings. We propose that these cells could be termed “dendritic brush cells” (DBCs). On the other hand, the current terminology is well established.

### Tbr2 Is Required for the Migration and Maturation of Unipolar Brush Cells

We previously found that UBCs/DBCs are produced from Tbr2^+^ progenitors in the rhombic lip (Englund et al., 2006; Pimeisl et al., 2013). In the present study we identified a genetic requirement for Tbr2 in timely migration from the rhombic lip and later expression of mature UBC markers. In the *Tbr2* cKO cerebellum many UBC/DBC precursors exhibited a migratory delay and clustered near the site of the former rhombic lip during periods of early postnatal development (Figure 8). Furthermore, there was no ectopic or aberrant expression of UBC markers, such as calretinin and mGluR1α, near the former rhombic lip or granular layer; rather, a deficiency of type I and type II UBCs/DBCs was observed throughout the *Tbr2* cKO cerebellum. We found no proliferative defects during the period of peak UBC genesis that would suggest cells were not being generated in this region at this key developmental time nor did we find abnormal rates of cell death in the cerebellum. From these results we infer that Tbr2 is required for the differentiation and timely migration of UBC/DBC precursors. However, we are unable to determine the ultimate fate of the Tbr2^+^ UBC progenitors in our *Tbr2* cKO cerebella: we observe no markers or morphology indicative of UBCs, no perturbations in cerebellar organization, and neither alterations of proliferation nor apoptosis appear to account for this population of cells. However, we will note that our analyses do not rule out the possibility of perturbations of progenitor proliferation at other developmental periods, and increased apoptosis of these cells may occur transiently, and/or very slowly, at times not observed in our studies.

Intriguingly, despite the lack of UBCs/DBCs no obvious locomotor phenotype such as ataxia has been observed in *Tbr2* cKO mice. Behavioral studies of *Tbr2* cKO mice have reported hyperactivity and reduced grip strength (Arnold et al., 2008) but neither we, nor others, have observed ataxia in *Tbr2* cKO mutants. In contrast, *moonwalker* mutant mice (*Trpc^–/–^*) are characterized by severe ataxia attributed to loss of type II UBCs (mGluR1α^+^ and PLCβ4^+^) as well as Purkinje cell dysmorphism and loss (Sekerkova et al., 2013). The *reeler* mouse, also ataxic, shows a defect in multiple cerebellar neuron types including UBCs/DBCs, which are reduced in number and ectopically positioned within the *reeler* cerebellum (Ilijic et al., 2005). Due to the multiple cerebellar neuronal populations affected in *moonwalker* and *reeler* mice, and overall complex and severe phenotypes of these mice, it is difficult to conclude that a lack of UBCs/DBCs – of either or both subtypes – causes ataxia in the absence of other abnormalities. Since UBCs/DBCs are most abundant in the vestibulocerebellum and dorsal cochlear nucleus, we hypothesize instead that *Tbr2* cKO mice may have specific problems with balance and auditory processing. These functions remain to be tested.

In cerebellar circuitry, the role of UBCs/DBCs as mossy fiber amplifiers suggests that they may be important modifiers but not critical general components of cerebellar circuitry. UBCs/DBCs are intermediate components in the cerebellar cortex that increase the excitatory influences of mossy afferent fibers on granule cells (Kalinichenko and Okhotin, 2005; Millen and Gleeson, 2008) and Purkinje cells (Ilijic et al., 2005). Thus, UBCs/DBCs function to enhance signals but are not a linchpin of the cerebellar circuitry. *Moonwalker* mice possess UBCs/DBCs during development and early postnatal periods of cerebellar development but begin to lose type II UBCs/DBCs at around one month of age (Sekerkova et al., 2013); *reeler* mice have a reduced number and ectopic localization of UBCs/DBCs (Ilijic et al., 2005). In contrast, *Tbr2* cKO mice show no evidence of normal UBCs/DBCs at any time during development in any region of the cerebellum. Thus, UBCs may not be critical for mouse locomotion and *reeler* and *moonwalker* ataxia may be due to the multiple cell populations affected and severely disorganized cerebella. In *reeler* mice, the cerebellum is extremely small and disorganized (Hevner, 2008), while *moonwalker* mice have, in addition to UBC loss, Purkinje cell loss that would cause ataxia (Sekerkova et al., 2013). An additional possibility is that the complete developmental lack of UBCs/DBCs in *Tbr2* cKO cerebella can be compensated, whereas the postnatal loss of the UBC/DBC element of cerebellar circuitry in *reeler* and *moonwalker* mice may contribute to the ataxic phenotype.

Unipolar brush cells are thought to confer an evolutionary advantage through an improvement in the vestibular system (Dino et al., 1999; Mugnaini et al., 2011). UBCs are most abundant in large animals that exhibit higher locomotor and visuomotor functions such as humans and non-human primates. Since mice are small animals, regarded as evolutionarily simple compared with other mammals, and there are numerous other differences between rodents and primates in multiple motor related pathways, the role of UBCs/DBCs in locomotion may be minimal in mice.

### Tbr2-Positive UBCs in the Developing Human Cerebellum

Because little is known about UBC number, localization, and function in humans, we examined Tbr2 expression during early periods of neurogenesis in the developing human cerebellum. In 19-week post-conceptional human cerebellum we observed a fountain-like migration of Tbr2^+^ cells from the rhombic lip with accumulations of UBCs/DBCs within the developing lobules X and IX. This migratory and colonization pattern are similar to the developing mouse cerebellum and suggests conservation of important lineage genes and migratory patterns in UBCs.

## Conclusion

UBCs/DBCs have complex morphology including multiple dendrites and brushes. Furthermore, UBCs/DBCs require Tbr2 for timely migration of UBC/DBC precursors from the rhombic lip into the granular layer and their differentiation and subsequent expression of mature markers including calretinin or mGluR1α.

## Data Availability Statement

The original contributions presented in the study are included in the article/supplementary materials, further inquiries can be directed to the corresponding author/s.

## Ethics Statement

The studies involving human participants were reviewed and approved by the Institutional Review Board (IRB) of Seattle Children’s Hospital. Written informed consent to participate in this study was provided by the participants’ legal guardian/next of kin. The animal study was reviewed and approved by Institutional Animal Care and Use Committee and the Seattle Children’s Research Institute.

## Author Contributions

AM, GE, and RH: conception and design of the study. AM, GE, RD, AB, DP, OD, and RH: acquisition and analysis of data. AM, GE, OD, and RH: manuscript and figure drafting. All authors reviewed the manuscript.

## Conflict of Interest

The authors declare that the research was conducted in the absence of any commercial or financial relationships that could be construed as a potential conflict of interest.

## Abbreviations

E: embryonic day
P: postnatal day
UBC: unipolar brush cell
cKO: conditional knock-out
KI: knock-in
DCN: deep cerebellar nuclei
NTZ: nuclear transitory zone.
